# A versatile toolkit for high throughput functional genomics with *Trichoderma reesei*

**DOI:** 10.1186/1754-6834-5-1

**Published:** 2012-01-02

**Authors:** André Schuster, Kenneth S Bruno, James R Collett, Scott E Baker, Bernhard Seiboth, Christian P Kubicek, Monika Schmoll

**Affiliations:** 1 Chemical and Biological Process Development, Energy and Environment Directorate, Pacific Northwest National Laboratory, 902 Battelle Blvd, Richland, WA, USA; 2 Research Area of Gene Technology and Applied Biochemistry, Institute for Chemical Engineering, Vienna University of Technology, Gumpendorfer Strasse 1a/1665, A-1060 Wien, Austria

**Keywords:** *Trichoderma reesei*; *Hypocrea jecorina*, transformation, vector construction, gene knock-out library, sexual crossing

## Abstract

**Background:**

The ascomycete fungus, *Trichoderma reesei *(anamorph of *Hypocrea jecorina*), represents a biotechnological workhorse and is currently one of the most proficient cellulase producers. While strain improvement was traditionally accomplished by random mutagenesis, a detailed understanding of cellulase regulation can only be gained using recombinant technologies.

**Results:**

Aiming at high efficiency and high throughput methods, we present here a construction kit for gene knock out in *T. reesei*. We provide a primer database for gene deletion using the *pyr4, amdS *and *hph *selection markers. For high throughput generation of gene knock outs, we constructed vectors using yeast mediated recombination and then transformed a *T. reesei *strain deficient in non-homologous end joining (NHEJ) by spore electroporation. This NHEJ-defect was subsequently removed by crossing of mutants with a sexually competent strain derived from the parental strain, QM9414.

**Conclusions:**

Using this strategy and the materials provided, high throughput gene deletion in *T. reesei *becomes feasible. Moreover, with the application of sexual development, the NHEJ-defect can be removed efficiently and without the need for additional selection markers. The same advantages apply for the construction of multiple mutants by crossing of strains with different gene deletions, which is now possible with considerably less hands-on time and minimal screening effort compared to a transformation approach. Consequently this toolkit can considerably boost research towards efficient exploitation of the resources of *T. reesei *for cellulase expression and hence second generation biofuel production.

## Background

The increasing awareness of the limited availability of fossil fuels along with the environmental problems caused by their application initiated considerable research efforts towards clean and sustainable biofuels [1-3]. Thereby, the cellulases required to degrade cellulosic plant materials into small building blocks, which can be metabolized by yeast or other microbes to ethanol or hydrocarbon biofuel precursors, respectively, are one major focus of investigation [4]. *Trichoderma reesei *(*Hypocrea jecorina*) is currently the most efficient producer of enzyme mixtures for degradation of plant materials [5]. The cellulases produced by this fungus are utilized for diverse industrial processes, from biobleaching of textiles, paper recycling to juice extraction and even as additives in animal feeds [6-8].

The long-standing use of cellulase production by *T. reesei *is paralleled by a thorough investigation of the cellulolytic enzyme system of this fungus and its regulation [9,10]. With publication of the genomic sequence of *T. reesei *[11], the progress in understanding the mechanisms of cellulase regulation was accelerated. Analysis of the genome indicated, that despite its production efficiency, *T. reesei *has the lowest amount of cellulolytic enzymes among Sordariomycetes at its disposal.

In order to gain an understanding of gene regulation, manipulation of the genome of *T. reesei *is indispensible. A transformation system for this fungus has been available for decades with *amdS *[12], *pyr4 *[13] and *hph *[14] being the most frequently used selection marker systems. Also pyrithiamine resistence [15], benomyl resistence [16] and hexokinase [17] have proven useful for transformation of *T. reesei*. Additionally, vector systems enabling excision of the marker gene cassette has facilitated multiple sequential genome modifications despite the limited availability of marker systems [18,19]. Nevertheless, the typical efficiency of homologous integration is less than 10% using these methods.

One of the most important advancements in recent years, for improving the performance of research with *T. reesei *was the development of strains deficient in non-homologous endjoining (NHEJ) [19,20]. These strains strongly enhance the probability of homologous integration of DNA constructs for deletion or modification of genes. Up to 95% of transformants were the result of homologous integration events [20]. While in most fungi little discernible phenotype is reported for this mutation, the respective strains are more sensitive to DNA damage [21], the HOG-MAPkinase pathway is up-regulated [22] and effects on genes involved in carbohydrate transport [23] have been observed in some organisms. The major drawbacks of using strains deficient in non-homologous end joining, however, is that this mutation causes telomere shortening and defects in DNA repair [21], both of which negatively influence genome stability and fitness of the respective strains.

Recently, a further tool for work with *T. reesei *became available: After decades of research leading to the conclusion that *T. reesei *is a clonal, asexual derivative of a previously sexual species [24], the capability of this fungus for sexual development was discovered [25]. Besides the physiological relevance, this finding also opens up a wide array of new possibilities for research with *T. reesei*. Crossing can now be used for strain improvement and classical genetics. However, the fact that the parental strain of all *T. reesei *strains used in research and industry, QM6a, is female sterile [25], necessitates the use of a sexually competent wildtype isolate for crossing. Consequently, the genetic background introduced by this closely related but also phenotypically different isolate represents a serious drawback for use of sexual crossing in research.

Genome sequencing and high throughput analysis methods for transcriptomics, proteomics and metabolomics have had considerable impact on how research with fungi progresses [26]. Despite considerable improvements in transformation techniques, the recent increase in available genomic sequences of fungi also caused the need to efficiently use these resources. However, despite the wealth of data created, in depth functional analysis of genes often lags behind and consequently many fungal genome databases still remain with their most precious treasures undiscovered. Mostly, this is due to the enormous effort necessary for creation of a gene knock out library, which necessitates thousands of experiments and subsequent screenings. Therefore the respective methods need to be streamlined and automated as much as possible. For the model organism, *Neurospora crassa*, a community-wide effort recently led to the completion of a whole genome knock-out library [27]. This resource is especially important for evaluation of transcriptome data, because screening of a large number of mutant strains considerably increases the knowledge and understanding to be gained from these studies [28,29]. Additionally, the possibility to study a group of functionally related genes, such as transcription factors can provide intriguing insights into previously unexplored physiological processes [30,31].

While elaborate molecular biological tools are available for other fungi, especially *N. crassa*, tools for efficient systematic analysis of gene function are relatively under-developed in *T. reesei*. Despite its widespread use in industry, no comprehensive gene knock out library is available for this fungus, nor are there currently efforts to start such an initiative. We therefore aimed to provide a dependable and easy to handle toolkit as a first step towards creation of larger sets of gene knock out strains in *T. reesei*.

In this study we explored strategies to enable high throughput gene-knockout along with efficient construction of multiple mutants. We applied yeast based recombination mediated vector construction, transformation by electroporation and subsequently removal of the NHEJ-deficient background by crossing with a sexually competent *T. reesei *strain derived from QM9414. Genome wide primer libraries for knock out vector construction using different marker systems complete this toolkit. These methods can serve as a basis for construction of large scale libraries of knock out strains for systematic functional genomic studies with *T. reesei*.

## Results and discussion

### Vector construction by yeast mediated recombination

**Figure 1:**
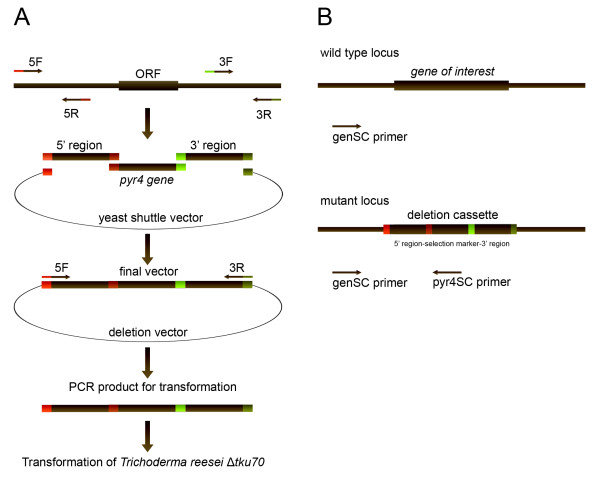
**(A) Schematic representation of vector construction by yeast mediated recombination**. (B) PCR based screening of putatively positive transformants.

The aim of this study was to enhance the efficiency of construction of deletion mutants to enable a high throughput approach for *T. reesei*. We therefore applied the method of yeast mediated recombination for deletion vector construction, which was shown to be highly efficient [30]. In order to test our approach we selected a set of 20 genes encoding proteins with a variety of predicted functions, such as transporters, transcription factors, peptaibol synthetases or G-protein coupled receptors (Table 1). The size of the predicted genes ranged between around 1000 to 4000 bp with the exception of the peptaibol synthetases which are larger than 50 kb. For amplification of the flanking sequences of these genes, primers were designed with the following properties: T_m_: 50 - 60°C, GC-content 40 - 60% and a length of 20 base pairs. We generated 5' flanking sequences and 3' flanking sequences with a size of around 1000 - 1500 bp's for each gene (Figure 1A, Additional file 1). In case of TR_23171 and TR_123786 (peptaibol synthetases genes with a size > 50.000 bp's) we chose as the 3' downstream flanking sequence a 1000 - 1500 bp's fragment from within the open reading frame. This selection should result in a deletion of the first 2200 base pairs of the gene and consequently in a non-functional peptaibol synthetase. As the selectable marker for *Trichoderma *transformation, we used the orotidine-5'-phosphate decarboxylase (*pyr4*, TR_74020) for transformation of *Δtku70 *which is also a uridine auxotroph [20]. The *pyr4 *gene was amplified from *T. reesei *wild-type DNA and used for yeast mediated recombination with the 3' and 5' flanking sequences containing 29 bp homologous overhangs into the shuttle vector pRS426 [30]. Total DNA was isolated from the yeast cells and was used as the template for amplification of the deletion cassettes by PCR.

**Table 1:** Overview about selected *T. reesei *genes.

protein ID	gene name	function	classification/group	ORF length [bp]	protein size [aa]	successful transformation method
2526	*pkc1*	protein kinase C	signal transduction	3888	1140	P
2845	*gna2*	g-protein alpha subunit two	signal transduction	1510	355	E
3873	*pde1*	hypothetical cyclic AMP phosphodiesterase, class II	signal transduction	2177	526	P
21505	*gna3*	g-protein alpha subunit three	signal transduction	1423	356	E
23171		nonribosomal peptide synthetase (NRPS)	secondary metabolism	69518	20874	N
28731	*gpr2*	hypothetical GPCR, family 2	G-protein coupled receptor	1258	374	E
37515	*rid1*	DNA methylase, required for RIP (GenBank Accession number: JN227866)	sexual development	1815	553	E
56684	*-*	sugar transporter	transport	1758	504	P
58456	*-*	Zn2Cys6 transcription factor	transcription factor	1589	445	P
65315	*-*	basic leucine zipper transcription factor	transcription factor	1046	299	P
70351	*-*	Zn2Cys6 transcription factor	transcription factor	1670	480	P
72004	*-*	hypothetical GPCR, family 2,	G-protein coupled receptor	1464	442	N
77795	*lim1*	putative E3 ubiquitin ligase	signal transduction	2004	637	E
79202	*-*	hypothetical transmembrane protein, transport protein	transport	1575	401	E
79756	*fwd1*	hypothetical F-Box/WD40 repeat protein	circadian rhythm	2832	920	P
102497	*-*	Zn2Cys6 transcription factor	transcription factor	2328	665	P
102655	*pde2*	hypothetical phosphodiesterase	signal transduction	2933	857	P
109088	*hpo1*	heterochromatin regulator	chromatin binding	1308	272	P
109146	-	PTH11-GPCR	G-protein coupled receptor	1604	452	P
123786	-	non-ribosomal peptide synthetase	secondary metabolism	50787	16535	P

Protein ID's were obtained from the *T. reesei *Database v2.0. (P: protoplast transformation, E: electroporation of *T. reesei *spores, N: not successful)

### Construction of deletion mutants in an NHEJ-deficient background

For high throughput gene deletion a high efficiency of homologous recombination is indispensable. Previous deletion of the *tku70 *gene in *T. reesei *TU-6, a uridine auxotrophic derivative of QM9414 [13], resulted in a strain deficient in non homologous endjoining resulting in a homologous integration efficiency which is higher than 95% [20]. For transformation we used both conventional protoplasting [13] and spore electroporation, as indicated with the respective genes (Table 1). Electroporation was found to be less time consuming and easier to handle,

From the resulting transformants we chose five colonies per gene, which considering the high recombination frequency should result in identification of a positive mutant. For screening we used a PCR based mutant screening. For each gene we designed a specific primer which binds outside from the deletion cassette in the flanking regions of the gene (Additional file 1 Figure 1B). In combination with a specific primer which binds inside of the selectable marker (*pyr4*) and the primer which binds outside from the cassette we were able to very efficiently screen the mutant strains by PCR. An integration of the deletion cassette at the homologous locus results in a specific amplicon with a size between 1400 to 2000 bp (data not shown) depending on the exact location of the outside primer.

The PCR screening method showed that we were able to generate 18 out of 20 deletion mutant strains. We failed with generation of mutant strains of the following genes: *tr_123806*, and *tr_72004*. The frequency of homologous integration was between 33 and 100%.

### Phenotype of *Trichoderma reesei *mutant strains

**Figure 2:**
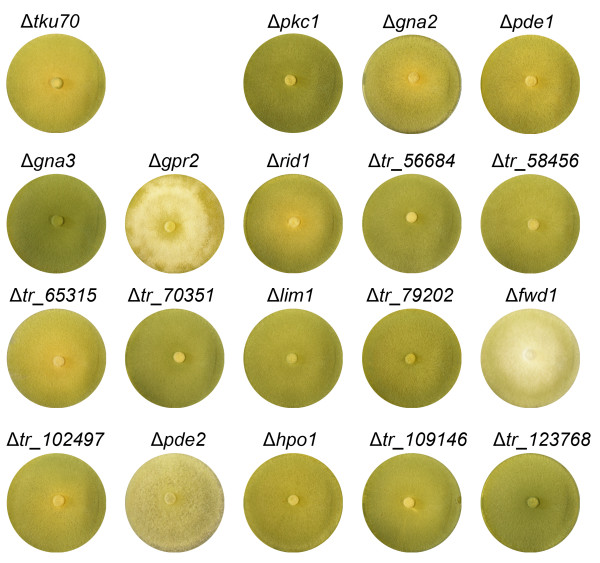
**Phenotypes of deletion mutant strains constructed in this study**. Strains were grown on malt extract agar plates for six days at 28°C.

The deletion mutant strains and the control strain (Δ*tku70*, supplemented with 1 M uridine) were cultivated on malt extract agar (3% (w/v) plates at 28°C for 6 days. We did not observe severe growth phenotypes for the strains studied, which corresponded well with the available *N. crassa *strains deleted for the predicted orthologous gene as available from the respective genome database http://www.broadinstitute.org/annotation/genome/neurospora/MultiHome.html (Figure 2).

The mutant lacking NCU00478.5, the homologue of the *T. reesei *phosphodiesterase gene *pde2 *(*tr_102655*), is white due to lack of pigment and no conidia are formed in slants on minimal medium at 25°C. Similarly, the respective *T. reesei *deletion strain Δ*pde2 *shows reduced growth and white pigmentation. Also the Δ*fwd1 *(*tr_79756*) strain shows reduced and white pigmentation which was also described for its *N. crassa *ortholog (NCU04540.5). Deletion of *gpr2 *(*tr_28731*) and *hpo1 *(*tr_109088*) in *T. reesei *leads to a reduced growth on rich medium but no phenotype was described for the orthologous genes (NCU04626.5 and NCU04017.5) strain in *N. crassa*. Deletion of *gna3 *(*tr_21505*) leads to a higher sporulation on malt extract plates, a phenomenon which was also shown for mutants of this gene in the closely related *Trichoderma atroviride *[32].

This pilot study showed that using vector construction by yeast mediated recombination, amplification of transformation cassettes and electroporation can significantly reduce hands-on time and considerably enhance efficiency of deletion mutant construction. We therefore went on to design an automated approach for construction of deletion primers for all *T. reesei *genes. This automated approach yielded a full set of primers for 8661 of the 9143 genes in *T. reesei *v2.0, as well as a partial set of primers for 488 of the genes for use with the *pyr4 *selection marker system in the NHEJ-deficient background of *T. reesei Δtku70 *(Additional file 2).

### Rescue of non homologous end joining by crossing

Since the deficiency in NHEJ not only brings the advantage of a higher probability of positive transformants, but also the problem of increased sensitivity to DNA damage, decreased genome stability and some changes in gene expression in some fungi [21-23], rescue of wild-type NHEJ is advisable.

**Figure 3:**
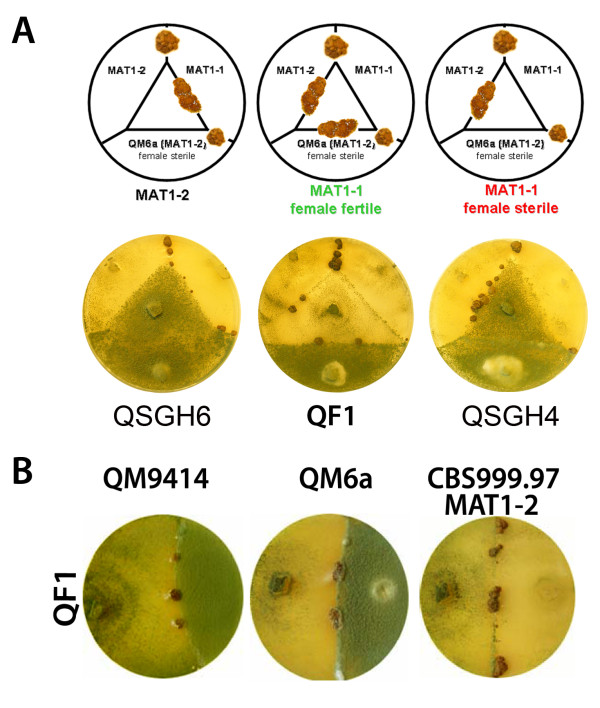
**Evaluation of strain properties for selection of a female fertile derivative of QM9414 with mating type MAT1-1**. (A) Parallel inoculation with test strains. CBS999.97 MAT1-1 and CBS999.97 MAT1-2 (designated as MAT1-1 and MAT1-2) as well as QM6a were inoculated around the outside of the plate and the strain to be tested (as indicated under the diagrams) in the center of the plate. Mating between CBS999.97 MAT1-1 and CBS999.97 MAT1-2 serves as positive control for sexual development. Fruiting body formation between MAT1-1 and QM6a serves as a positive control for testing female fertility of the respective strain. Fruiting body formation with QM6a is only possible if the tested strain has regained female fertility. A female fertile strain of MAT1-1 should form fruiting bodies with CBS999.97 MAT1-2 and the female sterile QM6a. Three strains with different characteristics are shown including the selected female fertile MAT1-1 strain QF1. QSGH6 has mating type MAT1-2 and QSGH4 is female sterile. (B) In order to confirm sexual competence of QF1 with strains used in research, QF1 was inoculated on a plate (left side) opposite of the strain to be tested (right side). The decreased conidiation of QF1 is a characteristic of the initiation of sexual development. (C) Phenotype of QM9414 and QF1. All strains shown in A-C were grown on malt extract medium.

The recently described sexual development of *T. reesei *[25] represents a new method for strain development in this fungus. It is possible with this technique to remove undesired mutations by crossing with compatible strains. For this purpose we created a sexually competent strain compatible with our mutants (dubbed QF1; MAT1-1) derived from QM9414 (MAT1-2). Therefore, progeny of a cross between the wild-type isolate *Hypocrea jecorina *CBS999.97 (MAT1-1) and the female sterile QM9414 were checked for mating type and sexual competence. Strains with MAT1-1 mating type which had gained female fertility were used for further crosses with QM9414. After several rounds of crosses, progeny were checked for desired properties by crossing with CBS999.97 MAT1-1 and CBS999.97 MAT1-2 in order to check for mating type and with QM6a to confirm female fertility (Figure 3A). A female fertile strain of mating type MAT1-1 was selected and checked for its ability to undergo sexual development with QM9414, QM6a and CBS999.97 MAT1-2 (Figure 3B). QF1 shows a similar phenotype as QM9414 as well as comparable cellulase production after five days of growth, albeit also CBS999.97 MAT1-1 produces cellulase levels similar to these strains (data not shown). Consequently we generated a strain with the retained capability of sexual development despite recovery of the QM9414 phenotype.

All mutant strains created in the course of this study are still able to mate with CBS999.97 and also with QF1 despite their deletions. As a proof of principle we selected three deletion strains for the crossing approach aimed at removal of the *tku70 *deletion: Δ*gna2*, Δ*gna3 *and Δ*rid1*. While Δ*gna2 *does not show a discernible phenotype, sporulation is increased in Δ*gna3 *and Δ*rid1 *is of particular interest, because in this mutant the mechanism of repeat induced point mutation (RIP) is likely to be perturbed. Deletion of the homologue of *rid1 *(RIP-deficient-1) in *N. crassa *abolishes RIP [33]. In *T. reesei *this phenomenon has not yet been demonstrated, nevertheless the low frequency of gene duplications in the genome of this fungus could be due to the operation of RIP [11].

Mating assays were performed on malt extract medium [25]. Crosses of these three strains were successful, albeit for Δ*gna3 *lower fertility in sexual crosses was expected [34]. After three weeks the resulting ascospores were isolated randomly and the colonies were grown on selective medium and analyzed. Growth on hygromycin containing medium indicated that the *tku70 *deletion was retained, and strains not able to grow in the presence of hygromycin were selected for further analysis. Growth on malt extract medium indicated presence of the initial gene deletion i. e. presence of the functional *pyr4 *marker gene. Strains growing on malt extract medium without uridine as supplement were checked for presence of the genes of interest. PCR screening confirmed restoration of the wild type *tku70 *and showed that the desired gene deletion was retained.

While this method provides a significant improvement compared to retransformation, because no additional marker genes are needed and the hands on time to achieve rescue of the deletion is decreased there are certain issues to be considered. Due to the number of backcrosses, QF1 is estimated to contain approximately 96 - 97% of the QM9414 genome including the same percentage of QM9414 specific mutations [35]. Thus, QF1 can also be assumed to contain 3 - 4% of the differences that distinguish QM9414 from CBS999.97.

Interestingly, a recent survey on classical mutations and genome polymorphisms in *N. crassa *[36] revealed significant genomic variation in classical mutant strains in this fungus. Nevertheless, these strains represent the basis for decades of research with *N. crassa *and the estimated level of difference between QF1 and QM9414 is well within the range used historically for *Neurospora *genetic studies.

The major difference between QF1 and QM9414 is likely to be found in the gene(s) involved in female fertility (and the genes located nearby in the genome), which is functional in QF1 and enables this strain to undergo sexual development with female sterile QM6a and its derivatives including QM9414. The nature of the responsible gene(s) is currently unknown as is its function. Additionally, QF1 has mating type MAT1-1 and hence certain differences in gene expression related to the mating type cannot be excluded [37]. We therefore recommend use of MAT1-2 progeny for further analysis and comparison with QM9414 data.

Although mating is a very efficient means of strain improvement and construction of multiple mutants, the fact that sexual development may introduce alterations in the genome has to be considered. In case of QF1, this includes the unlikely but possible interference of the small portion of the CBS999.97 genome retained in this strain. Therefore, segregation of the observed phenotype of a mutant with the selection marker has to be evaluated for several progeny. Additionally, as required with conventional transformations, different mutant strains have to be checked to confirm the effect of the mutation.

### Increasing utility of the system by additional selection markers

Since the mutation causing uridine-auxotrophy of Δ*tku70 *is not a complete deletion of the *pyr4 *gene [13], the preconditions for the operation of RIP (homologous sequences longer than 400 bps) would be fulfilled [38]. Use of alternative selection markers will likely prove to be beneficial. We consequently modified the full genome primer set described above for use with the *amdS *marker system [12], which can also be used with the NHEJ deficient strain Δ*tku70 *[20] (Additional file 3). Additionally, we provide this primer set for use with the *hph*-marker cassette (Additional file 4) used in the *N. crassa *high throughput gene knock out program [30] appropriate for transformation of the NJEH deficient strain QM6aΔ*tmus53 *[19]. These marker systems do not involve the use of homologous sequences of *T. reesei *and thus circumvent possible problems with RIP. Alternatively, Δ*rid1 *could be used for these constructions, which however would require subsequent removal of this background in order to avoid side effects by this mutation.

## Conclusions

The study presented here is intended to serve as a basis for fast and efficient analysis of gene function in *T. reesei*. Recent studies on genomes of early industrial mutants of *T. reesei *revealed several interesting mutations in these strains [35,39,40], but nevertheless, in many cases the specific genes responsible for the effect on cellulase gene expression as well as possible synergistic effects in these mutant strains, remain to be elucidated. Regulation of cellulase gene expression has been a focus of research with *T. reesei *for decades and hence a considerable number of transcriptome studies are available [41,42]. Since the availability of the genome sequence of *T. reesei *[11] several approaches using genome-wide microarrays have been reported [43,44]. These studies provide intriguing insights into various aspects of cellulase gene expression, from transcription factors to secretion pathways. Investigation of the target genes identified to be involved in cellulase regulation by analysis of gene knock out strains largely covering the involved pathways would be a logical further step towards elucidation of the mechanisms triggering induction, transcription, processing and secretion of cellulolytic enzymes.

The high relevance of *T. reesei *for the production of economically feasible second generation biofuels from cellulosic agricultural waste instead of food crops still requires considerable improvement of the efficiency of the cellulase mixture secreted [45]. Moreover, the use of *T. reesei *as a host for heterologous protein production necessitates a more detailed understanding of the physiology of this fungus. Our streamlined system for knockout-construction along with the convenient and easy to use method to create multiple mutants by crossing can serve as a starting point for large scale functional analysis studies aimed to uncover the hidden treasures of the biotechnological workhorse *T. reesei*.

## Methods

### Strains and plasmids

*T. reesei *Δ*tku70*, which is defective in the non-homologous end-joining pathway [20] was used in the present study and was maintained on potato dextrose agar (PDA, Difico, Detroit, USA) which was supplemented with 5 mM uridine (Sigma Aldrich, St. Louis, USA) in order to complement the auxotrophy of strain TU-6, a derivative of QM9414 [13]. Parental strain QM9414 (ATCC 26921; [35,46]) and strains derived from it were maintained on 3% (w/v) malt extract with 2% (w/v) agar 28°C. Mating experiments were performed as described previously [25]. Briefly, strains were grown on 3% (w/v) malt extract medium with 2% (w/v) agar. Plates were incubated at 20-25°C in daylight for 10 - 14 days until formation of fruiting bodies and ascospore discharge. For yeast transformation the shuttle vector pRS426 [47] and the yeast strain WW-YH10 (ATCC number: 208405) were used.

### Vector construction for gene deletion

For construction of deletion vectors for the selected genes (Table 1) the orotidine-5'-phosphate decarboxylase gene of *T. reesei *(*pyr4*, TR_74020) was used as selectable marker. The marker gene was amplified using primers pyr4F and pyr4R (supplementary file 1). The 50 μl reaction mixture contained 1.25 U Takara Ex Taq*™ *(Takara Bio, Madison, Wisconsin), 1 × Ex Taq*™ *Buffer, 0.2 mM dNTP, 0.1 μM forward and reverse primer, 1 μl *T. reesei *QM9414 genomic DNA (90 ng/μl) as template and nuclease free water. The selectable marker PCR fragment was purified using the E.Z.N.A. Gel Extraction Kit (Omega Bio-Tek, Inc., Norcross, USA).

For generation of the respective 5' and 3' flanking sequences, primers (Table 1) were designed with the OligoExplorer software (version 1.1.2; http://www.genelink.com/tools/gl-oe.asp). In order to enable yeast-mediated recombination of the deletion cassette, linker sequences were added to the primers for amplification of the 5' and 3' flanking region (supplementary file 1; Figure 1). Sequences were obtained from: *T. reesei *genome database (JGI webpage: http://genome.jgi-psf.org/).

The PCR mixture contained 1.25 U Takara Ex Taq*™ *(Takara Bio, Madison, Wisconsin), 1 × Ex Taq*™ *Buffer, 0.2 mM dNTP, 0.1 μM forward and reverse primer, 1 μl *T. reesei *wild-type genomic DNA (90 ng/μl) as template and nuclease free water.

### Database generation

A genome-wide database of primers for the 5' and 3' fragments for construction of the deletion cassette by yeast mediated recombination was generated using a Python script (provided by Peter Andrews of the *Neurospora *Genome Project) that serialized the function of the Primer3 core primer design program [48]. A masked genome sequence file and a gene annotation file for T. reesei v2.0 were downloaded from the Joint Genome Institute (JGI; http://genome.jgi-psf.org/Trire2/Trire2.download.html) and converted into a format suitable for input into the Python script. The default parameters which were used for Primer3 were the predefined 29 nucleotide sequences attached to the primers (supplementary file 1) and the following settings: optimal primer length = 20 bp, minimum primer length = 19 bp, flanking region length = 1500 bp, primer product size = min: 1000 bp and max.: 1300 bp, maximal size of GC clamp in primer = 2, primer optimal temperature = 56°C, primer GC content = min: 50% and max: 65%, monovalent catio salt concentration = 50 mM and concentration of annealing DNA oligo = 200 nM.

### Vector construction and yeast mediated recombination

The yeast shuttle vector pRS426 was digested with *Eco*RI and *Xho*I (restriction enzymes were purchased from New England Biolabs, Ontario, Canada) and purified with the E.Z.N.A. Gel Extraction Kit (Omega Bio-Tek, Inc., Norcross, USA). Yeast transformation and preparation was performed essentially as described previously [30,49,50].

An overnight culture (200 rpm, 30°C) of the yeast strain WW-YH10 was prepared. 1 ml of the overnight culture was added to 50 ml of fresh YPD (1% yeast extract, 2% peptone, 1% glucose (all chemicals from Sigma Aldrich, St. Louis, USA unless noted otherwise)) medium and incubated at 30°C until O.D.600 = 1. The cells were centrifuged at 14.000 rpm for 3 min. The supernatant was discarded and the pellet was washed with 25 ml of sterile water and centrifuged again for 3 min. The supernatant was discarded and the pellet was resuspended in 400 μl of 100 mM lithium acetate (Sigma Aldrich). 50 μl of yeast cell suspension was centrifuged for 20 sec and 240 μl 50% PEG 3550, 36 μl 1 M lithium acetate, 50 μl sheared salmon sperm DNA (2 mg/ml) (Sigma Aldrich) and 34 μl sterile water was added to the pelleted cells. Thereafter, 200 ng of 5' DNA fragment, 200 ng of 3' DNA fragment, 100 ng of digested pRS426 and 100 ng of selectable marker fragment was added and mixed. After the heat shock for 30 min at 42°C the mixture was centrifuged for 15 sec at 14.000 g and the supernatant was discarded. 1 ml of sterile water was added, and after thorough mixing centrifuged for 15 sec again. 800 μl of supernatant was discarded and the rest was spread onto SC-URA (1.54 g/l BSM-Iso-Ura Powder, 1.71 g/l yeast nitrogen base (Sunrise Science Products, San Diego, USA), 5 g/l ammonium sulfate, 20 g/l glucose and 20 g/l agar (Sigma Aldrich, St. Louis, USA)) plates and incubated for 3-4 days at 30°C. The resulting colonies were resuspended in 2 ml of sterile water and transferred into a new reaction tube. The sample was centrifuged for 15 sec at 14.000 g and the supernatant was discarded. 400 μl of smash-and-grab lysis buffer (2% Triton X-100, 1% SDS, 100 mM NaCl, 10 mM Tris pH 8.0, 1 mM EDTA), 500 μl phenol (pH = 7.8), 500 μl chloroform/isoamylalcohol (48:2) and 0.3 g of glass beads were added and mixed well. After centrifugation for 10 min at 14.000 g the supernatant was transferred to a new reaction tube and 500 μl of chloroform was added and mixed. The supernatant, after centrifugation for 10 min at 14.000 g, was transferred to a new reaction tube and 0.3 M sodium acetate (pH 5.2) and 2.5 volumes of ethanol (96%) were added. After precipitation and centrifugation for 10 min at 14.000 g, the pellet was washed with 400 μl 70% ethanol and resuspended in 50 μl of sterile water. The final linear deletion cassette was amplified from this solution by PCR. The mixture contained 1.25 U Takara Ex Taq*™ *(Takara-Bio, Madison, USA) appropriate buffer as described in the application manual, 0.2 mM dNTP, 0.1 μM forward (5F) and reverse primer (3R), 2 μl of isolated yeast DNA (approx. 100 ng) and nuclease free water. The protocol was: 94°C 3:00 min followed by 3 cycles of 94°C, 30 sec; 65°C, 30 s; 72°C, 2:00 min; followed by 2 cycles of 94°C, 30 sec; 63°C, 30 s; 72°C, 2:00 min; followed by 30 cycles of 94°C, 30 sec; 60°C, 30 s; 72°C, 2:00 min; and a final extension of 72°C, 10:00 min. The final linear deletion cassette was purified using the E.Z.N.A. Gel Extraction Kit (Omega Bio-Tek, Inc., Norcross, USA).

### Transformation of *T. reesei*

The *T. reesei *Δ*tku70 *strain [20] was used for transformation in order to achieve a high efficiency in homologous integration of the deletion cassettes. The protoplast transformation was carried out as previously described [13]. For transformation 2.5 μg of purified deletion cassette fragment was used. Transformants were grown on selective minimal medium (1 g/liter MgSO_4_*7H_2_O, 10 g/liter 1% KH_2_PO_4_, 6 g/liter (NH_4_)_2_SO_4_, 3 g/liter trisodium citrate*2H_2_O, 10 g/liter glucose, 20 ml/liter 50x trace elements solution (0.25 g/liter FeSO_4_*7H_2_O, 0.07 g/liter ZnSO_4_*2H_2_O, 0.1 g/liter CoCl_2_*6H_2_O, 0.085 g/liter MnSO_4_*H2O), 2% (wt/vol) agar; all chemicals were from Sigma Aldrich, St. Louis, USA).

The protocol for electroporation of *T. reesei *was adapted from patent application US2010/0304468. Spores of *T. reesei *Δ*tku70 *were harvested from a freshly sporulated 90 mm malt extract agar plate and suspended in 1.1 M sorbitol. Spores were washed twice, resuspended in 100 μl 1.1 M sorbitol and cooled on ice. 75 μl of cold spore suspension was mixed with 10 μg of vector of interest. For electroporation we used an Electroporation System ECM^® ^630 (BTX Instrument Division Harvard Apparatus, Holliston, USA). 1.8 kV, 800 Ω and 25 μF were used as setting for the ECM^® ^630 device. Thereafter one volume of the reaction mixture (consisting of spore suspension plus deletion cassette) of YEPD (1% (w/v) yeast extract, 2% (w/v) peptone, 1% (w/v) glucose) and four reaction volumes 1.1 M sorbitol were added and mixed. For regeneration the whole mixture was incubated over night at room temperature. Thereafter, spores were streaked out on plates containing selection medium.

Compared to the classical protoplast transformation technique [13] transformation by electroporation is less time consuming, easier to perform and the efficiency of this method was comparable to that of protoplast transformation.

Putative deletion strains were tested for integration of the construct by PCR using primer pyr4Sc (inside in the selectable marker gene *pyr4*) and the gene-specific primer, geneSc (outside from the transformation cassette) (supplementary file 1). Successful homologous integration results in a specific amplicon (1400 - 2000 bp's, depending on the gene) (supplementary file 1) and no amplification was possible with *T. reesei *Δ*tku70 *genomic DNA (data not shown).

### Construction of a sexually competent derivative of QM9414

The female sterile wild type strain QM9414 (MAT 1-2) and the sexually competent wild-type strain CBS999.97 (MAT1-1) [25] were inoculated on 3% (w/v) malt extract medium with 2% (w/v) agar for two to three weeks at 28°C until ascospore discharge. The ascospores were isolated from the lid with sterile water. This solution was spread out on a 3% (w/v) malt extract medium with 2% (w/v) agar plate and incubated at 28°C for two days until single colonies could be observed. The resulting colonies were screened for the mating type and again crossed to QM9414. The number of sexually competent strains decreased after each round of crossing, indicating a small region of the genomic to be relevant for this process. After five rounds of backcrossing, sexually competent strains with mating type MAT1-1 were tested for their phenotype upon growth on plates and a strain with characteristics largely indistinguishable from QM9414 was selected for further analysis.

For comparison of cellulase production, the parental strain QM9414 and QF1 were grown in 200 ml of liquid Mandels-Andreotti minimal medium with 1% (w/v) microcrystalline cellulose (No. 14204; Serva, Heidelberg, Germany) as carbon source at 200 rpm and 28°C in a rotary shaker. After 5 days of cultivation, samples were harvested and biomass formation and cellulase activity were measured as described previously [51].

### Crossing of the deletion mutant strains against QF1

The deletion strains were crossed with QF1 as described previously [25]. Ascopores were isolated and colonies were grown on selective medium in order to obtain progeny carrying the desired gene deletion, but not the Δ*tku70 *background. The resulting strains were tested for restoration of the *tku70 *locus using primers tku70ORFF and tku70ORFR. DNA was isolated with standard protocols. Successful restoration results in a specific band (480 bp's) for *tku70 *whereas no amplification was possible in the Δ*tku70 *background. Screening of mutants for propagation of the desired deletion was performed by PCR as described above.

## Competing interests

The authors declare that they have no competing interests.

## Authors' contributions

AS performed primer design, vector construction, transformation and screening of knock out strains and wrote the draft version of the manuscript, KSB and BS participated in conception of the study, KSB and and SEB supervised construction of knock out strains, JRC prepared the whole genome primer database, CPK and SEB conceived of the study, MS participated in conception of the study, suggested and constructed the backcrossed strain QF1, supervised work of AS and wrote the final version of the manuscript.

All authors have read and approved the final manuscript.

## Supplementary Material

Additional file 1 **Oligonucleotides used for vector construction and screening**. This file contains primer sequences, the respective fragment sizes of the amplicons prepared for vector construction along with the size of the amplicon for transformant screenings. Click here for file

Additional file 2 **Oligonucleotides for high throughput yeast mediated vector construction using the pyr4 marker system**. Click here for file

Additional file 3 **Oligonucleotides for high throughput yeast mediated vector construction using the amdS marker system**. Click here for file

Additional file 4 **Oligonucleotides for high throughput yeast mediated vector construction using the hph marker system**. Click here for file
